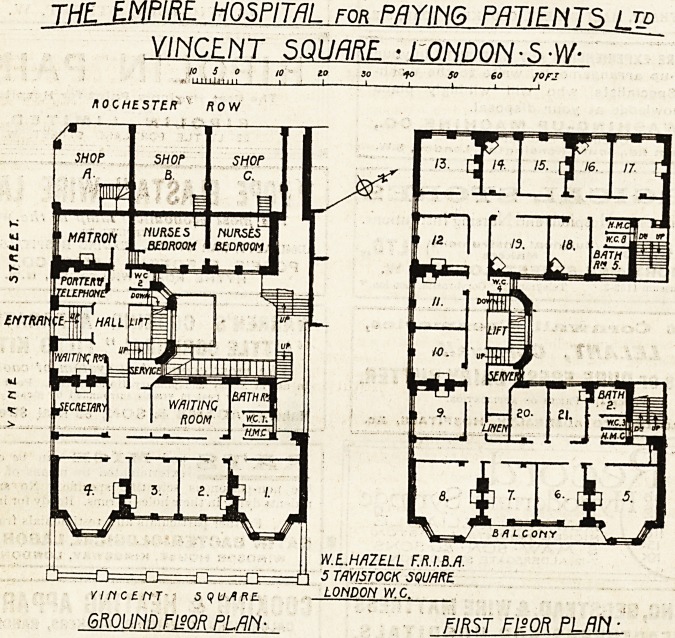# Empire Hospital for Paying Patients, Vincent Square, S.W.

**Published:** 1916-03-25

**Authors:** 


### HOSPITAL ARCHITECTURE AND CONSTRUCTION.

### Empire Hospital for Paying Patients, Vincent Square, S.W.

This building has been erected to accommodate some forty-five to fifty paying patients. The building has three frontages, and is planned round an inside courtyard about 30 feet by 24 feet. Four wards on each floor look on to this courtyard.

There can practically be no circulation of air in this courtyard, and we cannot but regard the rooms overlooking it as quite unfit for use as wards. The site, in fact, was unfitted for the purpose; for the fifty beds the site area is about 134 feet per bed, a figure which, compared with the average area of site per bed for general hospitals, seems absurdly inadequate. The fact appears to be that the building is planned more on the lines of a hotel than on those of a hospital, and is singularly unsuited for the latter purpose.

The sanitary offices all open directly into the corridors, and there does not appear to be any adequate provision for emptying and cleansing bed-pans and other utensils used in the wards. In one case the w.c. door is immediately opposite the door to a ward (No. 11).

Two operating theatres with anæsthetising room, sterilising room, and doctors' room are provided on the fourth floor, and a passenger lift is placed in the well of the staircase.

There are two fire-escape staircases at each end of the building.

The architect is Mr. W. E. Hazell, F.R.I.B.A.

## Figures and Tables

**Figure f1:**